# The ground beetles (Caraboidea) of the southern Sikhote-Alin Mountains

**DOI:** 10.3897/BDJ.9.e75509

**Published:** 2021-11-11

**Authors:** Yuri N. Sundukov, Kirill V. Makarov

**Affiliations:** 1 Federal Scientific Center of the East Asia Terrestrial Biodiversity Far Eastern Branch of the Russian Academy of Sciences, Vladivostok, Russia Federal Scientific Center of the East Asia Terrestrial Biodiversity Far Eastern Branch of the Russian Academy of Sciences Vladivostok Russia; 2 Moscow State Pedagogical University, Institute of Biology & Chemistry, Zoology & Ecology Department, Moscow, Russia Moscow State Pedagogical University, Institute of Biology & Chemistry, Zoology & Ecology Department Moscow Russia

**Keywords:** fauna, distribution, Russian Far East, Caraboidea

## Abstract

**Background:**

This paper presents the results of 30 years of field studies on the Caraboidea fauna of the southern Sikhote-Alin Mountain, Russian Far East. Material was collected at 300+ geographical localities within 18 administrative and seven urban districts of the Primorsky Krai, Russia. A total of 55,953 adult ground beetles belonging to 426 subspecies, 411 species, 86 genera and three families were studied. The families Rhysodidae and Trachypachidae are represented by one species each, while the family Carabidae the remaining 409 species. The resulting sampling-event dataset includes 12,852 occurrences.

**New information:**

This is the first dataset underlying an accurate and referenced taxonomic composition, as well as the geographic distribution of the Caraboidea in the southern Sikhote-Alin Mountains, Primorsky Krai, Russian Far East.

## Introduction

The fauna of ground beetles (Caraboidea) of the southern Sikhote-Alin Mountains is certainly one of the best studied in the Russian Far East. Until now, 427 species have been identified from this area (Lafer 1975, Lafer 1976a, Lafer 1976b, Lafer 1978a, Lafer 1978b, Lafer 1979, Lafer 1980, Kryzhanovskij 1983, Lafer 1989, Farkač and Plutenko 1992, Lafer 1992, Uéno and Lafer 1994, Moraveč and Wrase 1995, Uéno et al. 1995, Berlov and Berlov 1996, Farkač and Plutenko 1996, Fedorenko 1996, Lafer 1996, Matalin 1996, Kataev and Dudko 1997, Kataev and Jaeger 1997, Moraveč and Wrase 1997, Berlov and Berlov 1999, Hieke 1999, Sundukov 1999, Sundukov 2000, Sundukov 2001a, Sundukov 2001b, Sundukov 2001c, Hieke 2002, Sundukov 2003, Plutenko 2004, Sundukov 2004, Lafer 2005a, Lafer 2005b, Sundukov 2005, Dudko 2006, Sundukov 2006, Lafer and Kataev 2008, Sundukov 2008, Goulet et al. 2009, Sundukov 2009a, Sundukov 2009b, Sundukov 2009c, Shabalin and Lafer 2010, Sundukov 2010a, Sundukov 2010b, Sundukov and Smirnov 2010, Toledano and Schmidt 2010, Makarov and Sundukov 2011, Shabalin and Lafer 2011, Sundukov 2011a, Sundukov 2011b, Sundukov 2013, Sundukov 2019a, Sundukov 2019b, Sundukov and Makarov 2019, Sundukov and Makarov 2020, Sundukov and Sergeev 2021). However, information on the distribution of most species is very scant or completely absent. The main objective of our work was to reveal the taxonomic diversity of ground beetles (Coleoptera, Caraboidea) and their distributions in the territory of southern Sikhote-Alin. Most of the intensive fieldwork was carried out in 1993–2011, but the data were converted to the Darwin Core format in 2021.

## Project description

### Title

The ground beetles (Caraboidea) of southern Sikhote-Alin

### Personnel

Yuri Sundukov, Kirill Makarov

## Sampling methods

### Study extent

**Figure 1.**
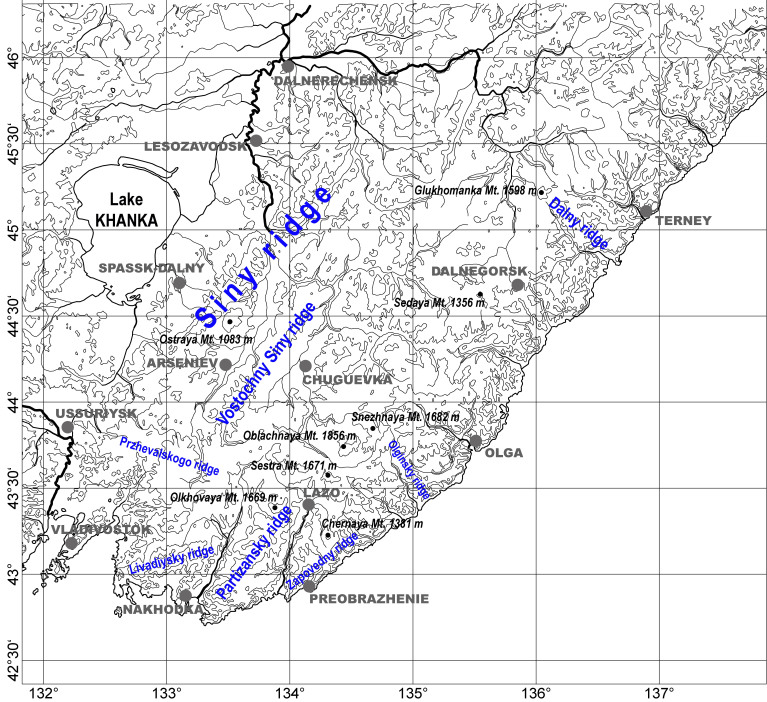
Schematic map of south Sikhote-Alin.

The available data are based on material collected during a 30-year long study of ground beetles in the south of Sikhote-Alin Mountains. During the period of the most intense explorations in 1993–2011, route and stationary studies covered a vast territory, ranging from the Muravyov-Amursky Peninsula in the south to the basins of Kema and Armu Rivers in the north (Fig. 1). Altogether, material was collected from more than 300 localities within 18 administrative and seven urban districts of the Primorsky Krai. Multiple expeditions were made to most of the high mountain peaks of the southern Sikhote-Alin. In addition to our own collections, the authors examined material taken by other researchers. As a result, a total of 55,953 adult specimens of Caraboidea were studied.

### Sampling description

When studying the Sikhote-Alin ground beetles, all available methods for their collection were used: hand-collecting , collecting with an exhauster, pitfall trapping, sweeping the crowns of trees and bushes, catching with conventional and ultraviolet light bulbs, night collecting with flashlight, "trampling" vegetation in humid and swampy habitats, water inundation in places at the edge of the water, sifting the litter with an entomological sieve, "mowing" on the grass with an entomological net, catching with a light trap, Malaise traps and window flight traps (Chapman and Kinghorn 1955, Hardwick 1968, Stewart and Lam 1968, Gillies 1969, Vanhercke et al. 1981, Kryzhanovskij 1983, Schauff 1986, Spence and Niemelä 1994, Dunaev 1997, Yahiro and Yano 1997, Skvarla et al. 2014). When working in stationary conditions, the collection was carried out using the various techniques as described above. During short trips and excursions, manual collection and pitfall trapping were mainly applied.

Our field research was carried out in the following landscapes and habitats:

1. Low mountains (intrazonal vegetation prevailing). They include the valleys of larger rivers, the sea coast and the peripheral regions of the Sikhote-Alin Mts. The fauna of low mountains is the richest in terms of taxonomy, but its component species show vast distributions and inhabit the entire territory as a rule (Sundukov 2000, Sundukov 2001b, Sundukov 2010a). There are some differences in the taxonomic compositions of the eastern and western macro-slopes of the Sikhote-Alin, since many boreal species penetrate much further south along the sea coast. To identify the species composition of this zone, it seemed sufficient to carry out field research at localities rather distant from one another.
2. Middle mountains (oak forests, cedar-broadleaved and dark coniferous forests are the most remarkable amongst the zonal vegetation communities). They take up the main part of the southern Sikhote-Alin area. The fauna is rich and includes species both widespread and numerous endemics and relics of various ranks (Berlov and Berlov 1996, Berlov and Berlov 1999, Sundukov 1999, Sundukov 2019a, Sundukov 2019b). When studying the fauna of the middle mountains, the latitudinal factor is of great importance: in the northern part of the southern Sikhote-Alin, species of the boreal complex take a significant part, whereas in the extreme south, species of the East Asian nemoral fauna predominate. For a sufficiently complete survey of this zone, research is required in at least three parts: northern, middle and southern.
3. Highlands (subalpine and alpine belts). In the south of Sikhote-Alin, such habitats are poorly developed, being represented by separated "islands" on tops of the highest mountains and ridges. Highlands support poor, but the most original faunas of ground beetles, including a large number of narrow endemics (Farkač and Plutenko 1992, Uéno and Lafer 1994, Farkač and Plutenko 1996, Sundukov 2001c, Sundukov 2009c, Sundukov 2010b, Sundukov 2019b, Sundukov 2019a). Surveying the southern Sikhote-Alin highlands must be carried out totally through visiting almost every peak towering above the upper timber line.

### Quality control

All collected specimens have been identified by the authors. The taxonomy and names of taxa are given in accordance with the Catalogue of Palaearctic Coleoptera (Löbl and Löbl 2017).

## Geographic coverage

### Description

The southern Sikhote-Alin is located in the extreme southeast of the mainland of Russia within the Primorsky Krai. According to the accepted physiographical zonation of the Far East, it occupies the southern part of the province of the Sikhote-Alin Mountains in the Amur-Primorsky landscape country (Parmuzin 1964, Ivashinnikov 2010). The surveyed territory is part of the vast mountainous Sikhote-Alin country lying between 42.8°–46.1° N and 131.8°–136.8° E (Fig. 1). Both from north to south and from west to east, the extent is about 400 km.

By its relief, the southern Sikhote-Alin is a typical mid-montane landscape. Its elevations average 600–1000 m above sea level, individual peaks up to 1600–1800 m a.s.l. (Oblachnaya, 1856 m a.s.l.; Snezhnaya, 1682 m a.s.l.; Sestra, 1671 m a.s.l.; Olkhovaya, 1669 m a.s.l.). The highest peaks are sharply outlined, being covered with stony screes over vast areas as a rule. In addition to the main watershed ridge, the orographic composition of the southern Sikhote-Alin includes seven almost parallel ridges stretched mainly from southwest to northeast along the coast of the Sea of Japan (Fig. 1). In the extreme south, these are the Przhevalskogo, Livadiysky, Partizansky and Zapovedny mountain ridges. To the north, along the eastern border of the Khanka Lowland, there are the Siny, Vostochny Siny and Kholodny mountain ridges. In addition to these, in the east, there are the Olginsky and Dalny latitudinal ridges.

In the southern Sikhote-Alin, forest vegetation prevails, occupying about 97% of the territory (Petropavlovsky 2004). By origin, three forest groups can be distinguished: successional (communities formed in river floodplains) (Fig. 2a, b), virgin (represented only by fir-spruce forests of the upper mountain belt and alpine vegetation) (Fig. 2c, d, e) and derivatives (various derivative coniferous-broadleaved forests, prevailing in the south of Sikhote-Alin) (Figs 2f, 3a, b) (Taran 2002). Forestless vegetation types include: coastal littoral (Fig. 3c, d), meadows (Figs 3e, f, 4a), bogs (Fig. 4b), subalpine shrubs (Fig. 4d), mountain meadows (Fig. 4c) and tundra (Fig. 4e, f), all occupying small areas. A characteristic feature of the vegetation of the southern Sikhote-Alin is a well-pronounced zonation which is due to the elevation above sea level, the geomorphological structure of the surface and the influence of the sea.

### Coordinates

42.59 and 46.133 Latitude; 131.619 and 137.937 Longitude.

## Taxonomic coverage

### Description

The dataset (Sundukov and Makarov 2021) includes information on 96 subspecies and 411 species of the superfamily Caraboidea, all belonging to 86 genera, 32 tribes, 13 subfamilies and three families (Table 1). In total, we have found 96.25% (411 of the 427) species known from southern Sikhote-Alin (Sundukov 2013).

## Traits coverage

### Data coverage of traits

PLEASE FILL IN TRAIT INFORMATION HERE

## Temporal coverage

### Notes

1929-06-14 through 2021-08-13

## Collection data

### Collection name

Federal Scientific Center of the East Asia Terrestrial Biodiversity FEB RAS (collection of Y Sundukov)

### Specimen preservation method

DRIED

## Usage licence

### Usage licence

Creative Commons Public Domain Waiver (CC-Zero)

## Data resources

### Data package title

The ground beetles (Caraboidea) of southern Sikhote-Alin

### Resource link

http://gbif.ru:8080/ipt/resource?r=sikhotecarab

### Alternative identifiers

https://www.gbif.org/fr/dataset/f0633e1c-1b2d-4d80-b881-065e5de44897

### Number of data sets

1

### Data set 1.

#### Data set name

The ground beetles (Caraboidea) of southern Sikhote-Alin

#### Data format

Darwin Core

#### Number of columns

34

#### Character set

UTF-8

#### Download URL

https://www.gbif.org/occurrence/download?dataset_key=f0633e1c-1b2d-4d80-b881-065e5de44897

#### Description

The dataset includes the results of long-term studies of the Caraboidea fauna of the southern Sikhote-Alin (Primorsky Krai, Russian Far East). The data are based on the collections of the authors during 1990–2011 at numerous locations in this mountainous region. In addition, information about collection material collected in other years or received from other collectors, which are stored in the collection of the author, is included. In total, the dataset include information on 55953 specimens of adults of ground beetles belonging to 411 species from 86 genera and three families of Caraboidea. They are distributed between families as follows: Rhysodidae - one species, Trachypachidae - one species and Carabidae - 409 species.

The dataset consists of one table Occurrence with 33 columns. The fields include the scientific name and the number of specimens, descriptions of habitats, geography and date.

**Data set 1.** 

Column label	Column description
basisOfRecord	Preserved Specimen (in all tables)
class	Insecta (in all records)
continent	Asia (in all records)
coordinateUncertaintyInMetres	The horizontal distance (in metres) from the given decimalLatitude and decimalLongitude describing the smallest circle containing the whole of the Location
country	Russian Federation (in all records)
countryCode	Country code, RU in all records
county	Full, unabbreviated name of the next smaller administrative region than stateProvince in which the Location occurs.
day	The integer day
decimalLatitude	The geographic latitude
decimalLongitude	The geographic longitude
eventDate	The date or interval during which an Event occurred
family	Full scientific name of the family in which the taxon is classified (Carabidae, Trachypachidae or Rhysodidae)
genus	Generic name
geodeticDatum	Geodetic datum, WGS84 in all records
habitat	Category or characteristic of the habitat in which the beetles are collected
kingdom	Animalia (in all records)
locality	The specific description of the place
month	The integer month
occurrenceID	An composite identifier for Occurrence: the first 4 letters of the generic epithet, 10 letters from the specific epithet and the ordinal number of the entry for this species
order	Coleoptera (in all records)
organismQuantity	A number value for the quantity of specimens
organismQuantityType	The type of quantification system used for the quantity of organism
phylum	Arthropoda (in all records)
recordedBy	A person, responsible for recording the original Occurrence
scientificName	The full scientific name, including author and year
specificEpithet	The name of the first or species epithet of the scientificName
stateProvince	Primorsky (Maritime) Kray, in all records
taxonRank	The taxonomic rank of the most specific name in the scientificName (species in all records)
verbatimCoordinates	The verbatim original spatial coordinates
verbatimCoordinateSystem	In all tables: degrees minutes seconds
verbatimEventDate	The verbatim original representation of the date information
verbatimLocality	The original textual description of the place
year	The four-digit year
institutionCode	FEB (Federal Scientific Center of the East Asia Terrestrial Biodiversity RAS) in all cases

## Additional information

Sundukov Y, Makarov K (2021). The ground beetles (Caraboidea) of southern Sikhote-Alin. Version 1.2. Moscow Pedagogical State University (MPGU). Occurrence dataset https://doi.org/10.15468/ebx56x accessed via GBIF.org on 2021-08-27.

## Figures and Tables

**Figure 2a.**
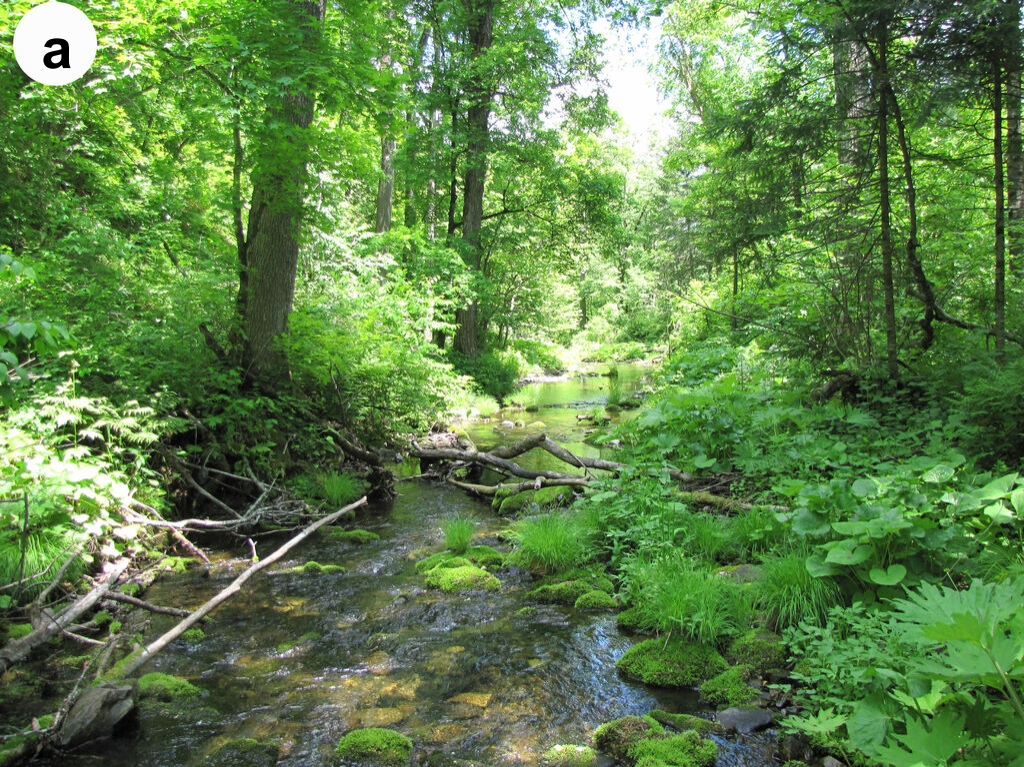
floodplain chozenia-poplar forest, 2nd Log Stream

**Figure 2b.**
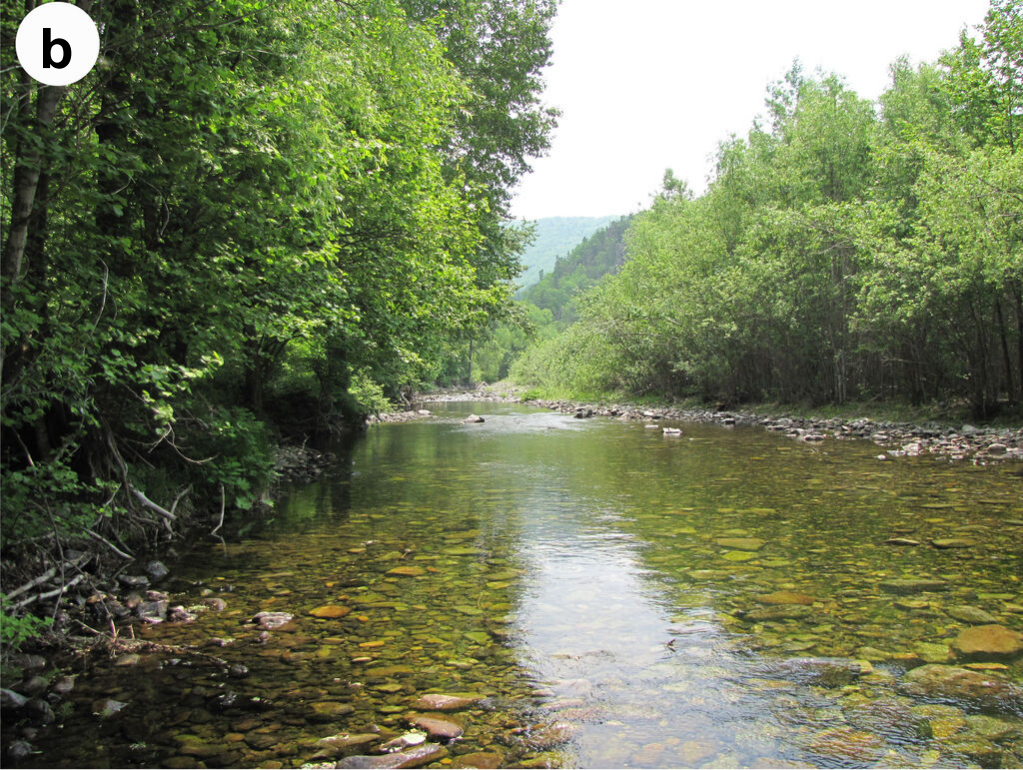
floodplain alder-willow forest, Perekatnaya River

**Figure 2c.**
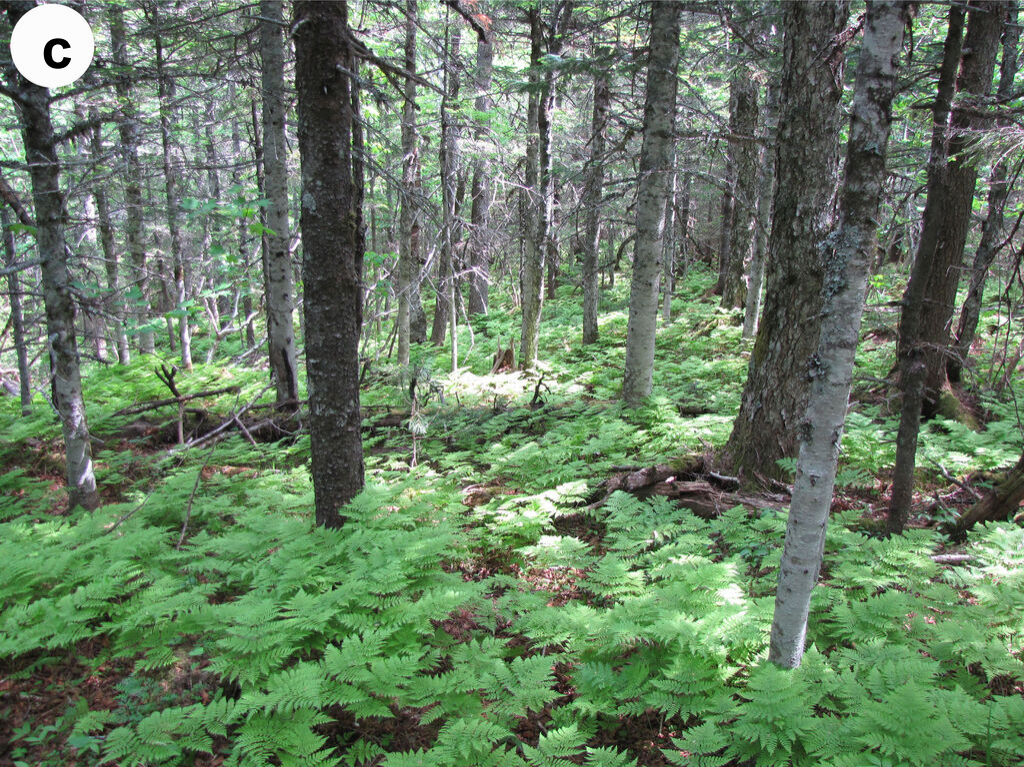
dark coniferous fern forest, Snezhnaya Mt., 1400 m a.s.l.

**Figure 2d.**
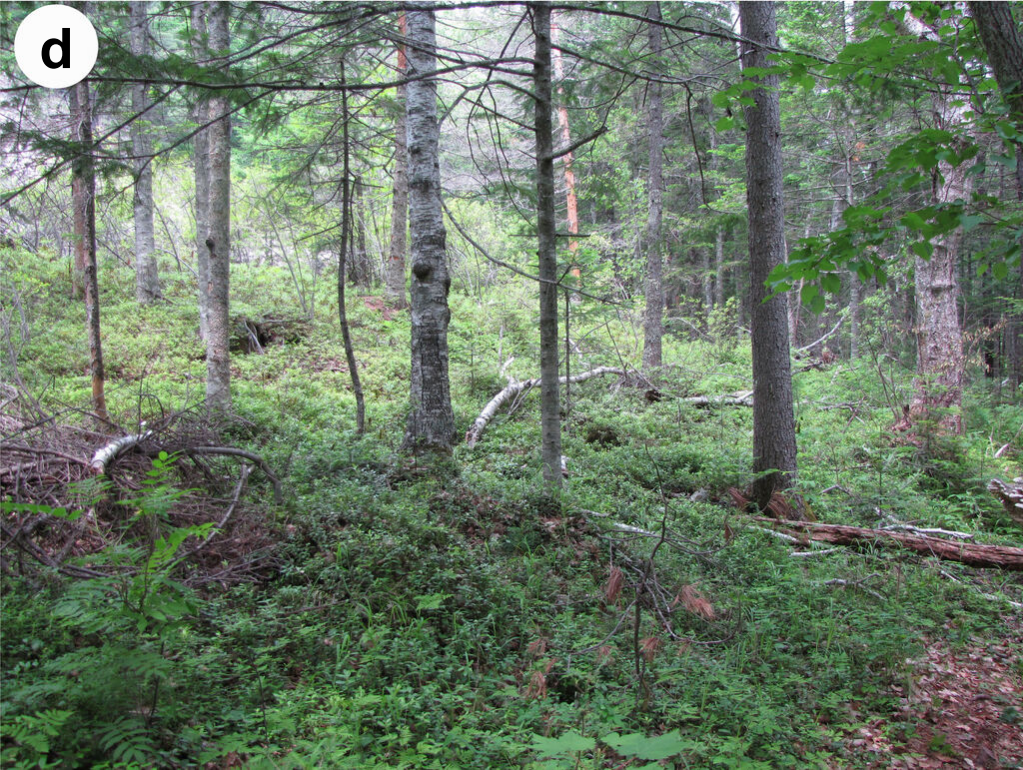
dark coniferous lingonberry forest, Chernaya Mt., 1200 m a.s.l.

**Figure 2e.**
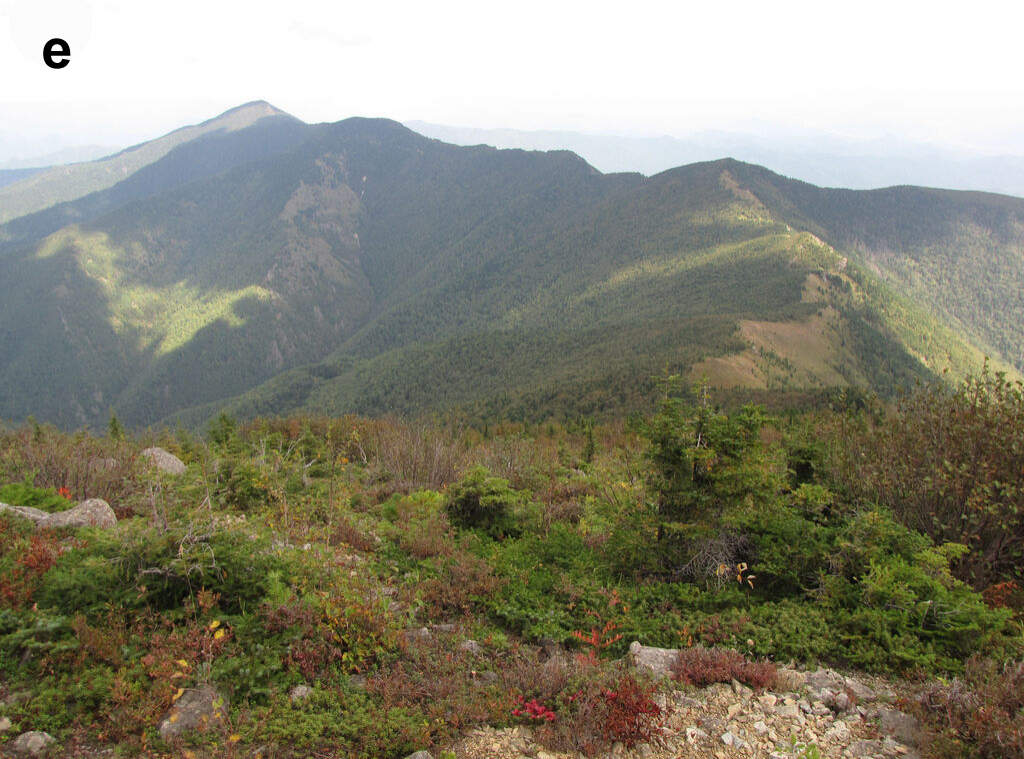
alpine woody vegetation, Krutaya Mt., 1650 m a.s.l.

**Figure 2f.**
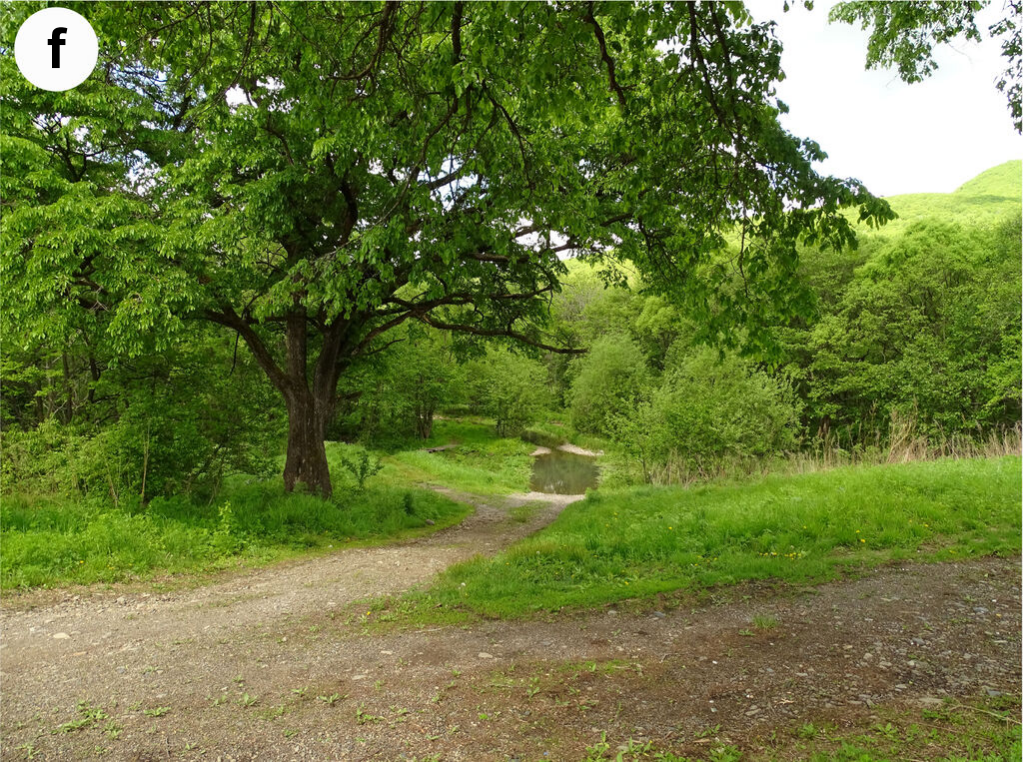
valley broadleaved forest, Lazo environs.

**Figure 3a.**
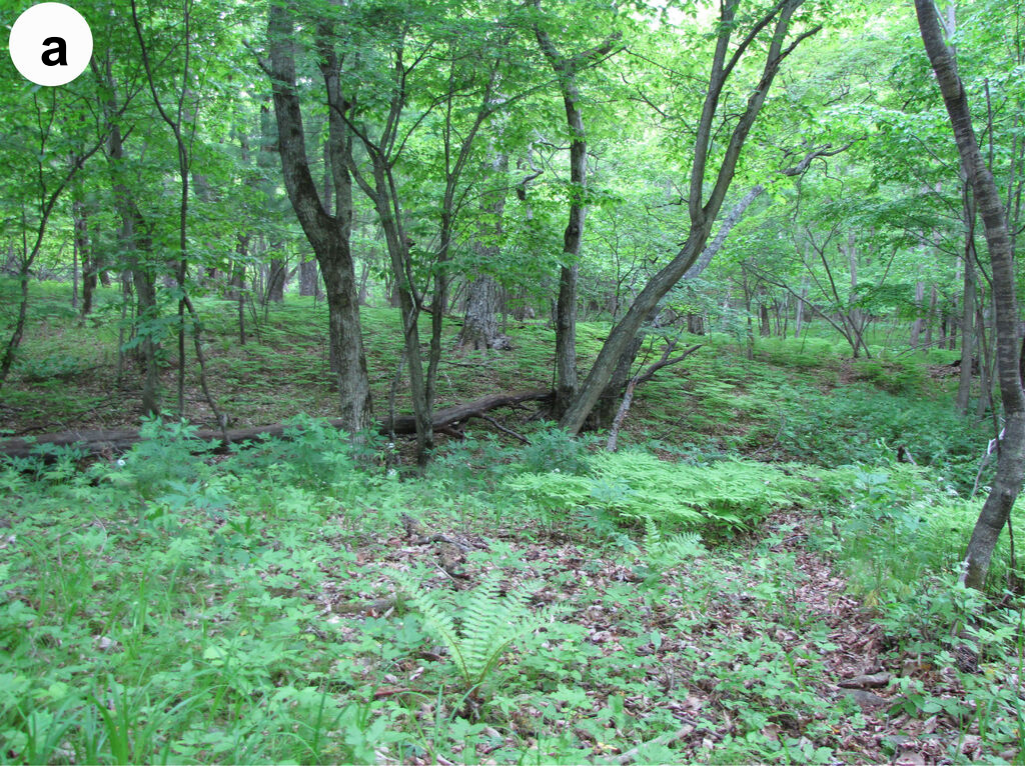
valley cedar-broadleaved forest, Sukhoi Klyuch Stream

**Figure 3b.**
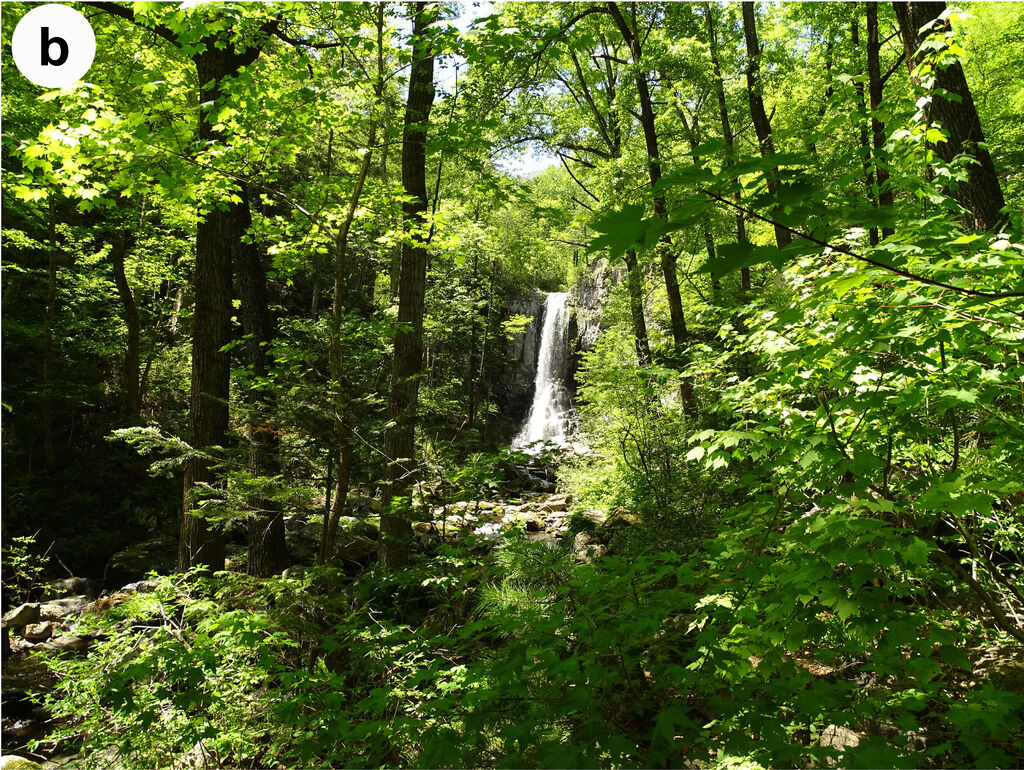
mixed coniferous-deciduous forest, Elamovsky Stream, 650 m a.s.l.

**Figure 3c.**
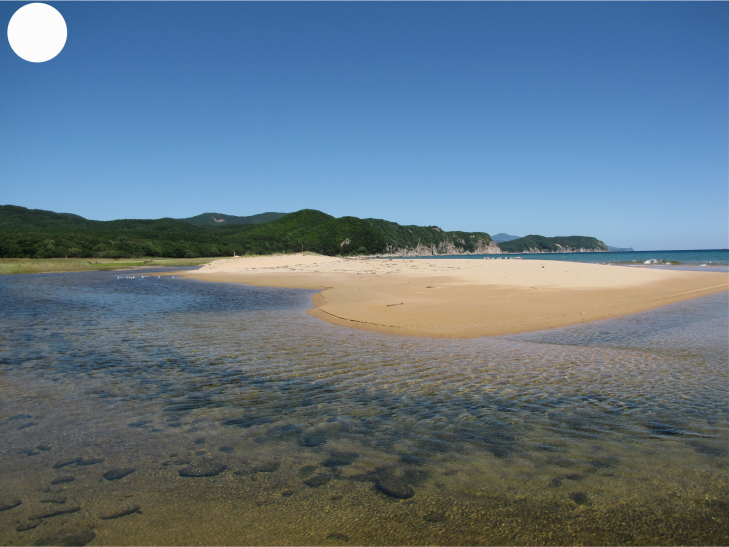
sea coast, mouth of Proselochnaya River

**Figure 3d.**
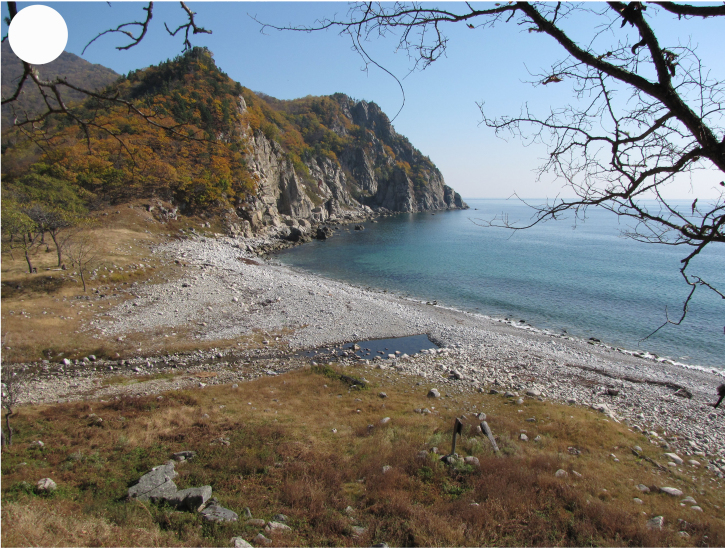
sea coast, Uglovaya Bay

**Figure 3e.**
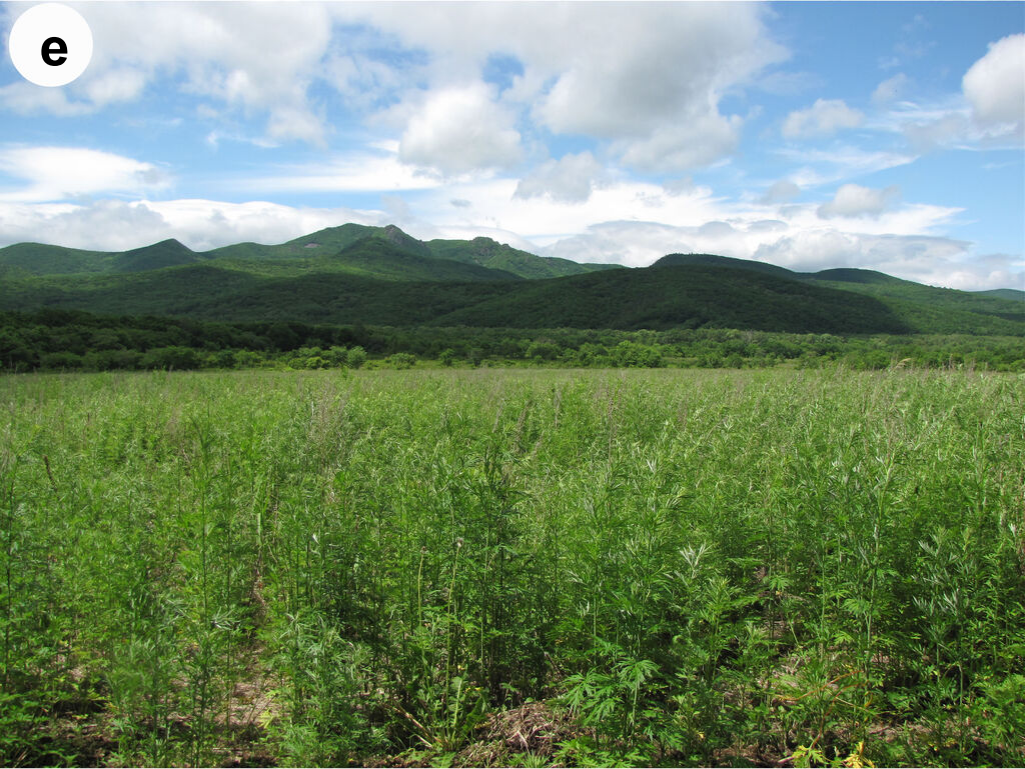
overgrown arable land, valley of Polyarnaya Zvezda River

**Figure 3f.**
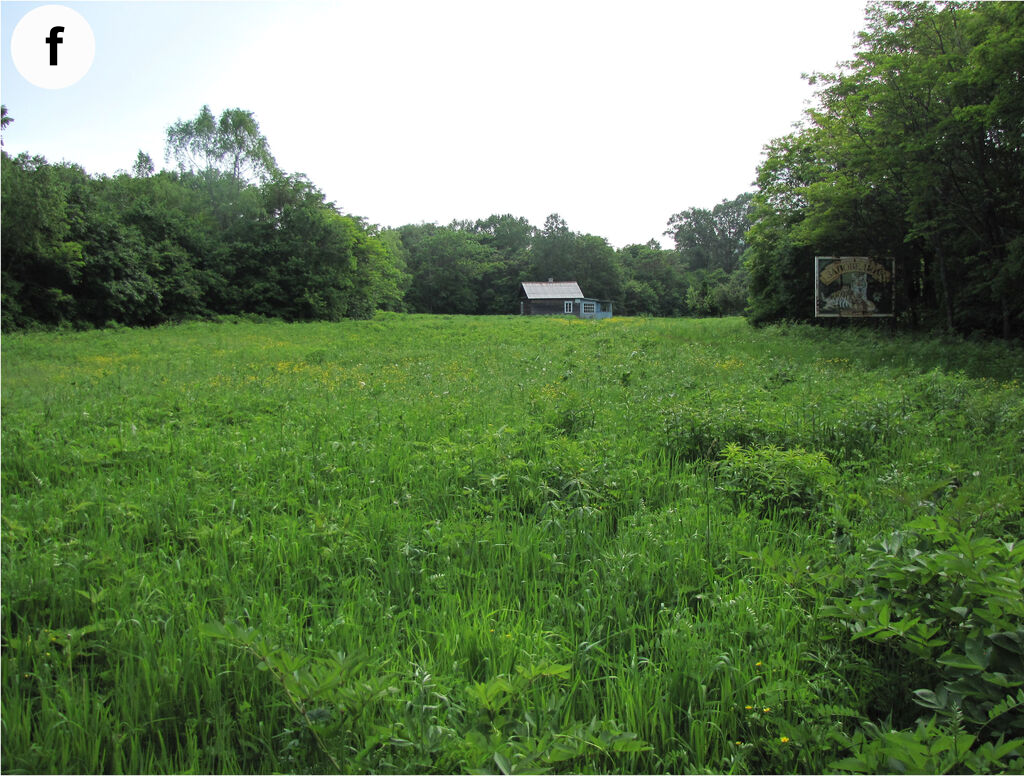
glade in valley forest, America tract.

**Figure 4a.**
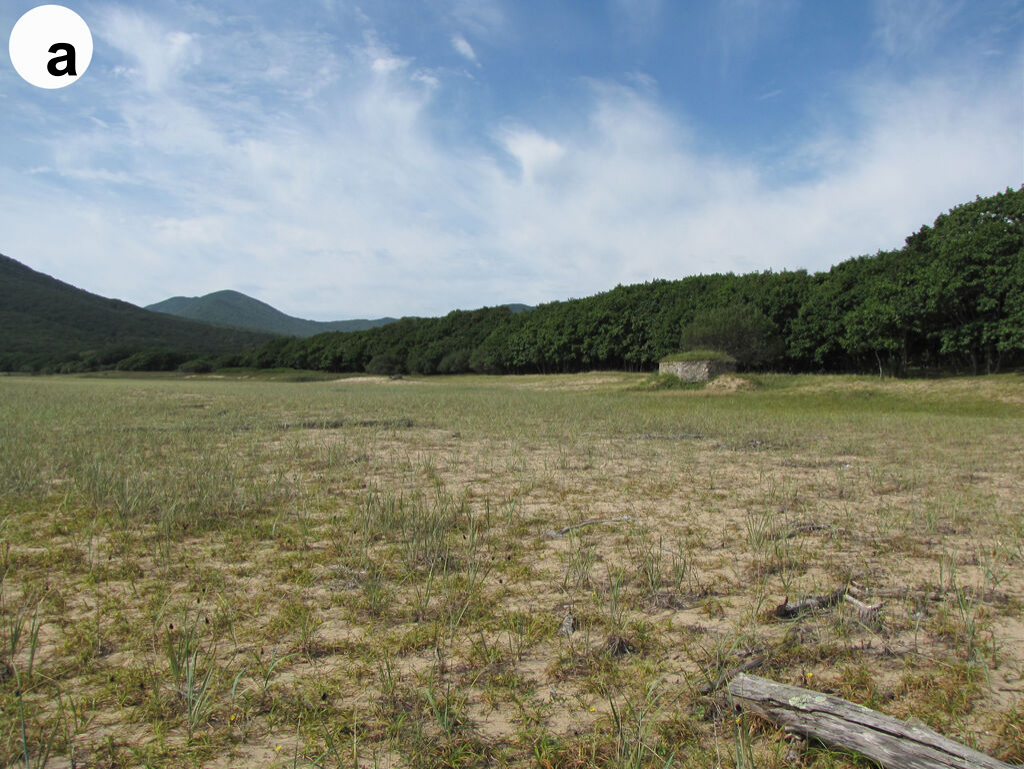
seaside sandy meadow, Proselochnaya Bay

**Figure 4b.**
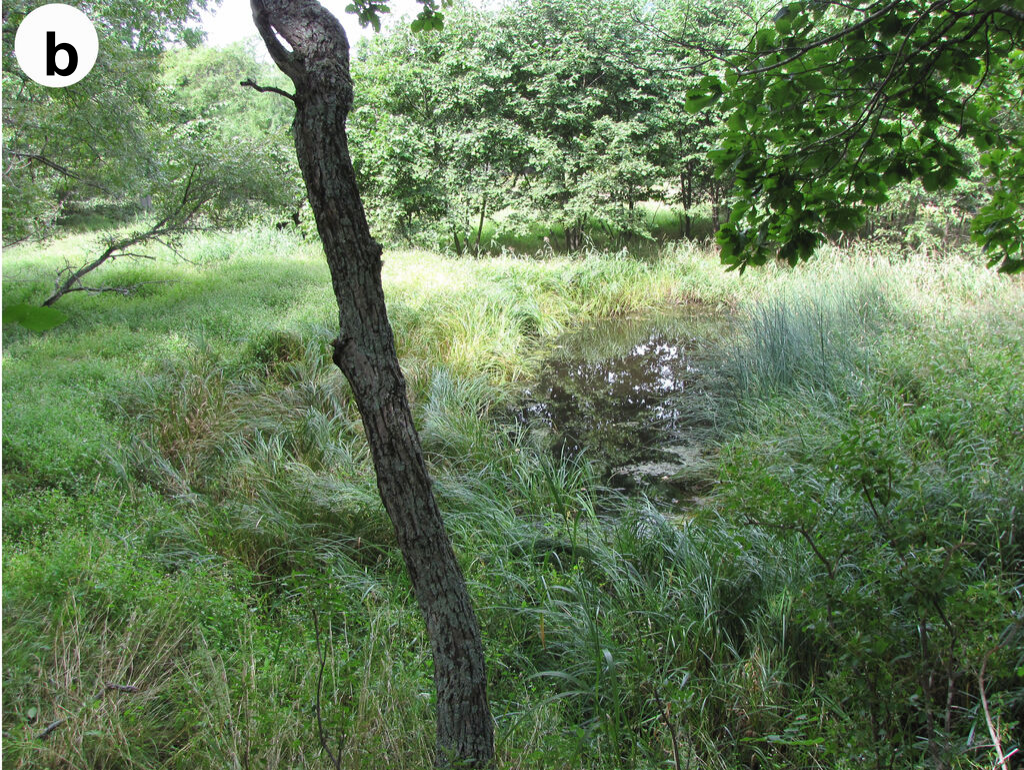
floodplain sedge bog, Sokolovskaya Bay

**Figure 4c.**
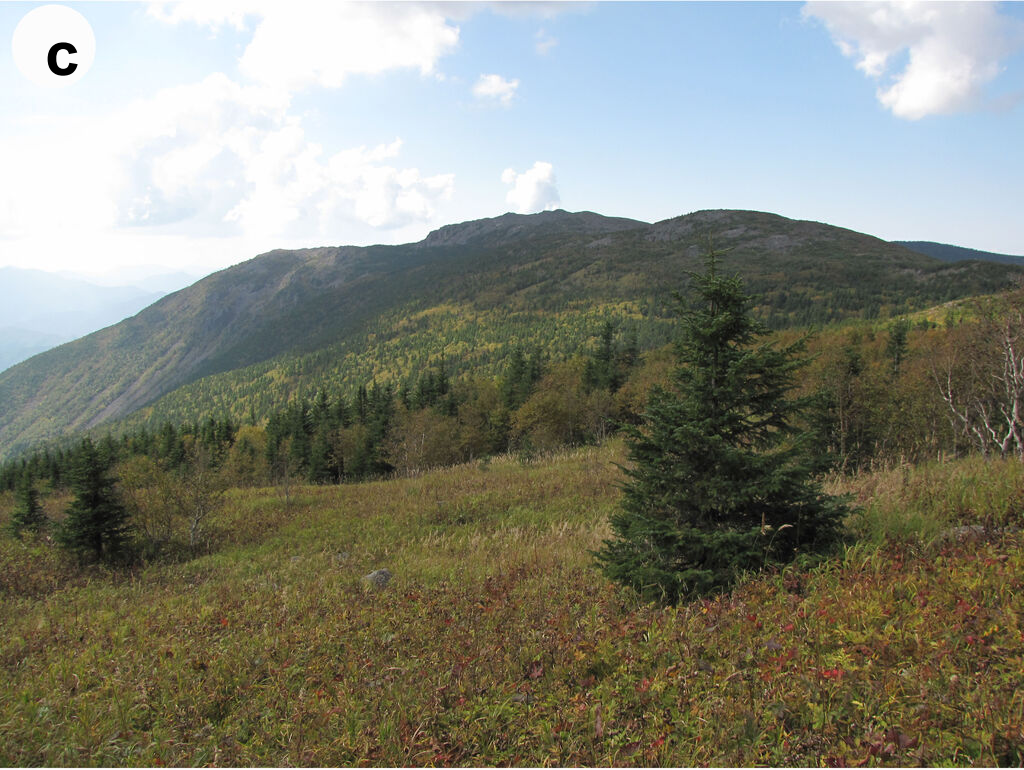
subalpine meadow, Olkhovaya Mt., 1200 m a.s.l.

**Figure 4d.**
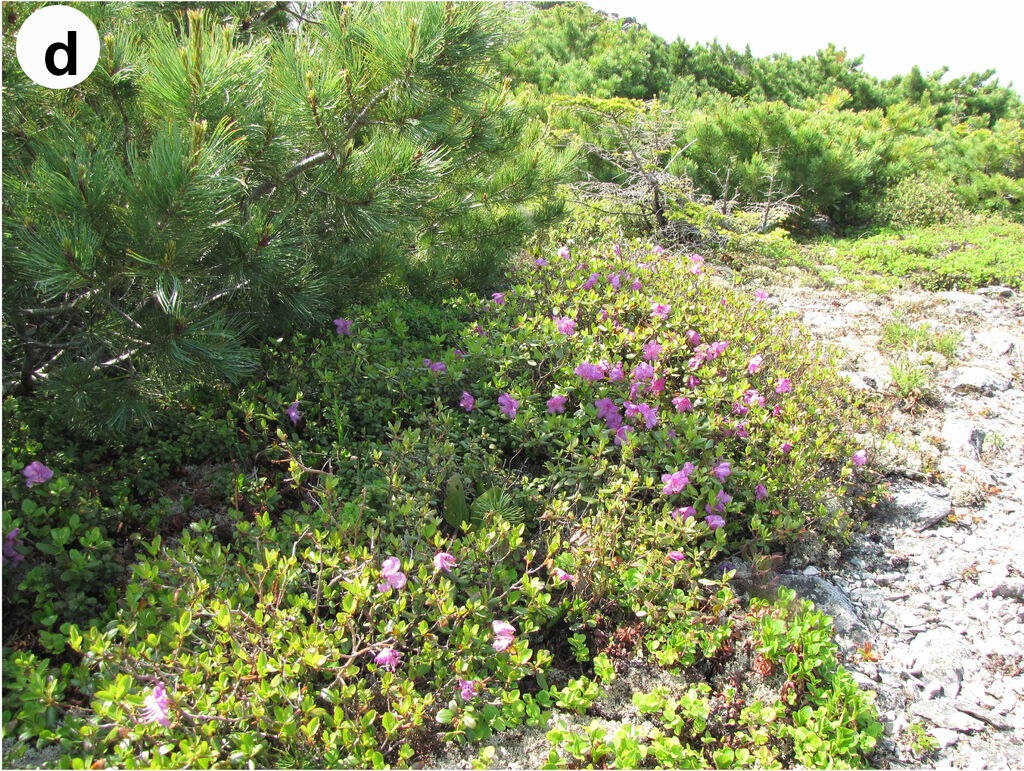
alpine shrubs, Oblachnaya Mt., 1600 m a.s.l.

**Figure 4e.**
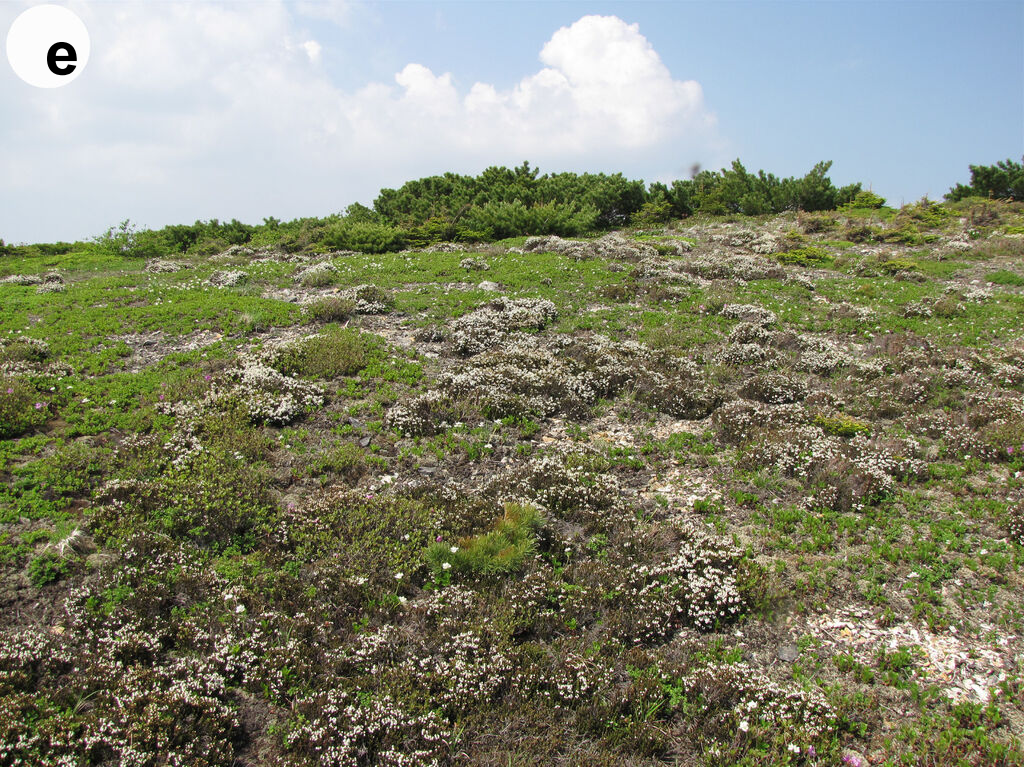
mountain tundra, Oblachnaya Mt., 1700 m a.s.l.

**Figure 4f.**
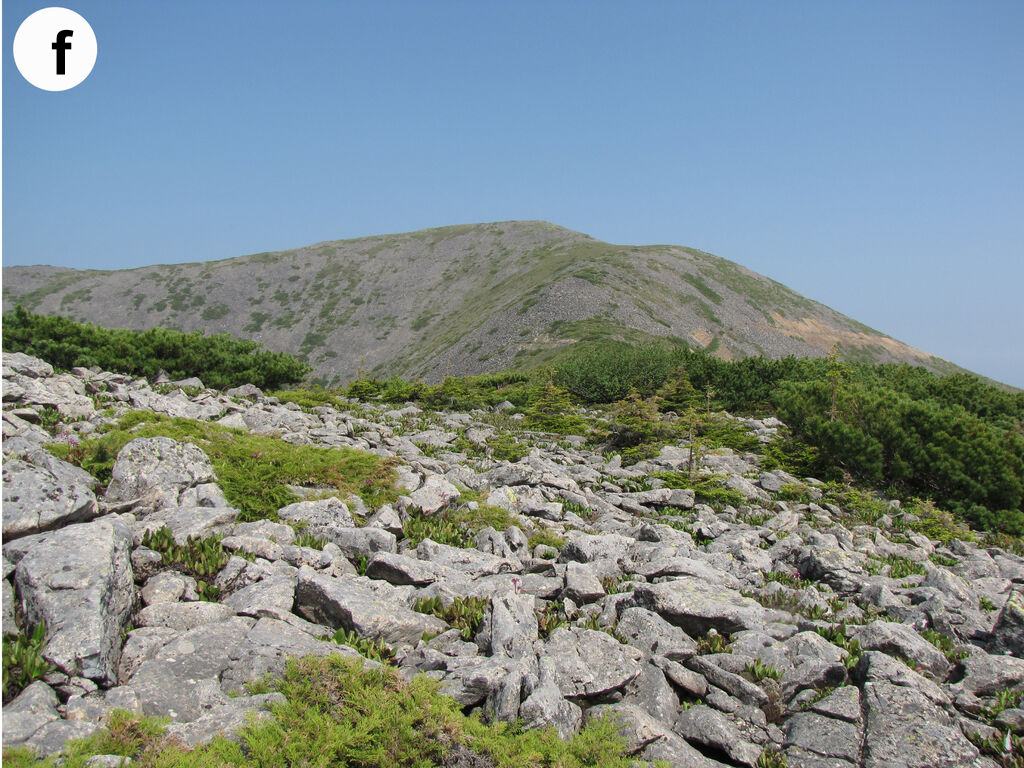
rocky tundra, Snezhnaya Mt., 1500-1680 m a.s.l.

**Table 1.** Taxonomic diversity of Caraboidea in the southern Sikhote-Alin.

Family	Tribe	genera	species	subspecies	total taxa	locations	specimens
Carabidae	Bembidiini	2	56	13	56	2,156	11,750
Carabidae	Brachinini	2	3	1	3	40	362
Carabidae	Broscini	3	3	0	3	16	92
Carabidae	Carabini	2	19	21	24	1,040	5,208
Carabidae	Chlaeniini	1	11	1	11	220	778
Carabidae	Cicindelini	2	7	6	7	145	363
Carabidae	Clivinini	1	2	1	2	8	14
Carabidae	Cychrini	1	1	1	1	41	74
Carabidae	Dryptini	1	1	0	1	32	58
Carabidae	Dyschiriini	1	9	5	9	91	188
Carabidae	Elaphrini	1	6	0	6	61	256
Carabidae	Harpalini	9	56	6	56	1,738	5,563
Carabidae	Lebiini	12	29	2	29	640	2,208
Carabidae	Licinini	3	9	2	9	105	309
Carabidae	Loricerini	1	1	1	1	7	7
Carabidae	Nebriini	2	13	4	13	474	2,014
Carabidae	Notiophilini	1	4	0	4	156	393
Carabidae	Odacanthini	1	2	1	2	8	11
Carabidae	Omophronini	1	1	1	1	10	72
Carabidae	Oodini	2	3	0	3	37	119
Carabidae	Panagaeini	2	3	1	3	33	39
Carabidae	Patrobini	2	3	0	3	123	568
Carabidae	Pentagonicini	1	2	0	2	9	9
Carabidae	Perigonini	1	1	0	1	5	9
Carabidae	Platynini	11	31	2	31	800	2,872
Carabidae	Pterostichini	2	51	17	58	2,420	11,705
Carabidae	Sphodrini	3	13	2	13	458	2,343
Carabidae	Tachyini	4	6	2	6	237	1,696
Carabidae	Trechini	8	18	6	21	317	2,713
Carabidae	Zabrini	1	45	0	45	1,395	4,076
Rhysodidae	Rhysodini	1	1	0	1	22	75
Trachypachidae	Trachypachini	1	1	0	1	8	9
**Total**		**86**	**411**	**96**	**426**	**12 , 852**	**55 , 953**
